# Facile doping of nickel into Co_3_O_4_ nanostructures to make them efficient for catalyzing the oxygen evolution reaction†

**DOI:** 10.1039/d0ra00441c

**Published:** 2020-03-31

**Authors:** Adeel Liaquat Bhatti, Umair Aftab, Aneela Tahira, Muhammad Ishaq Abro, Muhammad Kashif samoon, Muhammad Hassan Aghem, Muhamad Ali Bhatti, Zafar HussainIbupoto

**Affiliations:** Institute of Physics, University of Sindh Jamshoro 76080 Sindh Pakistan; Department of Science and Technology, Campus Norrköping, Linköping University SE-60174 Norrköping Sweden; Institute of Chemistry, University of Sindh 76080 Jamshoro Pakistan zaffar.ibhupoto@usindh.edu.pk; Mehran University of Engineering and Technology 7680 Jamshoro Sindh Pakistan; Centre of Pure and Applied Geology University of Sindh Jamshoro 76080 Sindh Pakistan; Centre of Environmental Sciences 76080 Jamshoro Sindh Pakistan

## Abstract

Designing a facile and low-cost methodology to fabricate earth-abundant catalysts is very much needed for a wide range of applications. Herein, a simple and straightforward approach was developed to tune the electronic properties of cobalt oxide nanostructures by doping them with nickel and then using them to catalyze the oxygen evolution reaction (OER) in an aqueous solution of 1.0 M KOH. The addition of a nickel impurity improved the conductivity of the cobalt oxide, and further increased its activity towards the OER. Analytical techniques such as scanning electron microscopy (SEM), energy dispersive spectroscopy (EDS) and powder X-ray diffraction (XRD) were used to investigate, respectively, the morphology, composition and crystalline structure of the materials used. The nickel-doped cobalt oxide material showed randomly oriented nanowires and a high density of nanoparticles, exhibited the cubic phase, and contained cobalt, nickel and oxygen as its main elements. The nickel-doped cobalt oxide also yielded a Tafel slope of 82 mV dec^−1^ and required an overpotential of 300 mV to reach a current density of 10 mA cm^−2^. As an OER catalyst, it was shown to be durable for 40 h. Electrochemical impedance spectroscopy (EIS) analysis showed a low charge-transfer resistance of 177.5 ohms for the nickel-doped cobalt oxide, which provided a further example of its excellent OER performance. These results taken together indicated that nickel doping of cobalt oxide can be accomplished *via* a facile approach and that the product of this doping can be used for energy and environmental applications.

## Abstract 2

Designing a facile and low-cost methodology to fabricate earth-abundant catalysts is very much needed for a wide range of applications.

## Graphical abstract

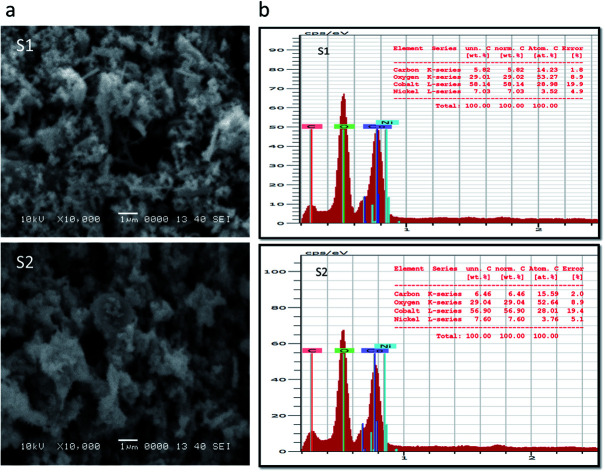

## 1. Introduction

In current times, the energy crisis resulting from the depletion of fossil fuels is a key problem for people around the world. The frequent use of fossil fuels has seemed unavoidable,^1^ but has harmed our environment due to this use resulting in emissions of carbon and resulting global warming.^2^ The economy of every country depends on using energy either for industrial, domestic, and/or transportation purposes.^3,4^ Fortunately, we have alternates to fossil fuels. We can get clean forms of energy that are not harmful to the environment from renewable energy resources. The resources of renewable energy are essentially endless and of low cost.^5–8^ They include solar, wind, tidal, geothermal, hydrogen or oxygen fuel cells, *etc.*^9–15^ But one of the deficiencies of these sources of energy is that they are not easily available in all areas of the world. For example, the intensity and timing of solar energy is different in different countries, wind speed is not always suitable for wind turbines in certain areas, and several countries lack sea and river assets used for producing energy from tides, waves, and currents.

Water splitting^16–19^ is a very familiar technique to scientists. It involves use of the oxygen evolution reaction (OER) and hydrogen evolution reaction (HER).^19–22^ Both reactions are used for eco-friendly energy production. The energy used for the OER is produced in various ways, such as solar, wind and nuclear technologies.^23–25^

Having at hand an efficient electrocatalyst is one of the requirements for successfully performing the OER. Noble metal catalysts such as Ru/Ir-based materials are the state-of-the-art catalysts for the OER but they are very costly and rare in nature. Alternative nonprecious catalysts are, therefore, being sought. Also, the applicability of the OER is limited due to its high overpotential. An ideal OER catalyst must have a low overpotential and must be stable. The OER is a naturally slow reaction, so we need to use catalysts to lower the energy barrier and hence increase the rate of the reaction.^26–30^ In the recent past, extensive studies have been carried out on the utilization of earth-abundant materials for the OER such as metal oxides including cobalt oxide,^31–34^ nickel oxide,^35–37^ manganese oxide,^38,39^*etc.*

Cobalt oxide has emerged as an interesting metal oxide for its significant catalytic activity toward the OER. Cobalt oxide is a relatively inexpensive and earth-abundant material.^40–42^ The efficiency of cobalt oxide for the OER has been summarized in the literature.^40–42^ Cobalt oxide doped with tungsten has been found to be an efficient OER catalyst.^43–48^ Due to the highly corrosive nature of cobalt oxide in alkaline media and its intrinsically poor conductivity, it performs poorly for the OER.^49^ Different strategies related to nickel incorporation into cobalt oxide have been used, but they are expensive and complicated.^50,51^ Therefore, facile and cost-effective approaches are needed to incorporate nickel into cobalt oxide for practical applications. Despite the progress made in the OER field, we still need to improve the efficiency of catalysts based on cobalt oxide by improving its electrical conductivity and preventing its corrosion in alkaline media. Therefore, for this purpose, we have added an impurity of nickel into cobalt oxide, and found this addition to yield an efficient, durable, and stable nonprecious OER catalyst. The nickel impurity might alter the electronic features of cobalt oxide, and it did yield an increase in the catalytic activity and conductivity of cobalt oxide and consequently made its OER performance more efficient.

Here, we present a simple approach for the design of an outperforming OER catalyst, by doping nickel into cobalt oxide. The nickel-doped cobalt oxide nanostructures were characterized using scanning electron microscopy (SEM), powder X-ray diffraction (XRD), and energy dispersive spectroscopy (EDS). The OER activity was strongly affected by the addition of nickel into cobalt oxide in alkaline media. The catalyst was observed to require an overpotential of 300 mV to reach a current density of 10 mA cm^−2^. Also the catalyst was found to be durable for 40 h and remained stable. Furthermore, an EIS study revealed that the doped materials displayed rapid charge transport, explaining their superior OER activity levels.

## 2. Experimental methods

Metal salts such as cobalt chloride hexahydrate, urea and nickel chloride hexahydrate were purchased from Sigma Aldrich, Karachi Pakistan. The synthesis of the nickel-doped cobalt oxide was done in two steps. For the synthesis of nanostructured materials, the aqueous chemical growth method was used and an equimolar concentration (0.1 M) of cobalt chloride hexahydrate and urea was mixed in 100 mL of deionized water. Two cobalt precursor solutions were prepared in separate beakers, to which 75 and 100 mg of nickel chloride hexahydrate, respectively, were added. These two samples were labelled S1 and S2. Afterwards, the beakers were covered with aluminum foil and left in a pre-heated electric oven for 5 h at 95 °C. After the completion of the reaction, the nanostructured materials were collected *via* filtration using a common laboratory filter paper. Then the samples were left to dry overnight at 60 °C and annealed at 500 °C in air for 5 h. This process yielded the nickel-doped cobalt oxide product ready to be used for further experiments. A similar method was used to prepare pristine cobalt oxide without the addition of nickel impurity.

The nanostructured materials were characterized by performing SEM at an accelerating voltage of 15 kV, and powder XRD was used to determine the internal structure and phase purity of the produced cobalt oxide materials. To determine the atomic percentage of each element in the composite sample, we acquired its EDS spectrum.

### 2.1. Electrochemical measurements for OER characterization

The electrochemical measurements for the OER were taken by carrying out linear sweep voltammetry (LSV) and cyclic voltammetry (CV) in 1.0 M KOH. We used a three-electrode cell system. A platinum wire was used as the counter electrode and silver with silver chloride (Ag/AgCl) filled with 3 M KCl was used as the reference electrode. Various working electrodes were tested, each one composed of a glassy carbon electrode (GCE) modified with a different catalyst: in each of these cases, first a mass of 5 mg of the catalyst was dispersed in a mixture of deionized water (2.5 mL) and 5% Nafion (500 μL); and after stirring the mixture, a volume of 10 μL of the resulting catalyst ink was dropped on a GCE following the drop casting method, and the resulting electrode was dried at room temperature. Chronopotentiometry was used to investigate the durability of each catalyst at a constant current density of 10 mA cm^−2^. Electrochemical impedance spectroscopy was also performed for the frequency range 100 kHz to 0.1 Hz at an amplitude of 10 mV and onset potential of 1.53 V *vs.* the reversible oxygen electrode (RHE) in 1.0 M KOH electrolyte. Z-View software was used to analyze the EIS data. The reported potentials were against the reversible hydrogen electrode (RHE). The overpotentials were calculated by subtracting RHE from the thermodynamic potential 1.23 V.

## 3. Result and discussion

### 3.1. Physical characterization of the nanostructured materials

**Fig. 1:**
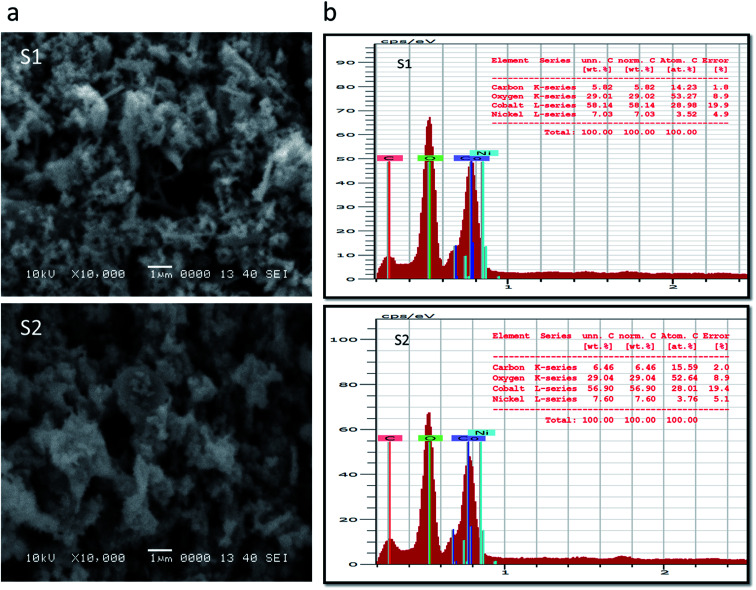
(a) SEM images of S1 and S2. (b) EDS spectra of S1 and S2.

Inspection of the SEM image acquired for sample S1 indicated the presence of a mixture of nanowires and nanoparticles, as shown in Fig. 1a. The nanowires were each a few microns in length and 100–200 nm in diameter, and the nanoparticles showed dimensions of 100–150 nm. However, in the case of S2, upon increasing the content of nickel salt, the morphology is changed into clusters of aggregated nanoparticles, also shown in Fig. 1a. Here, the dimensions of nanoparticles were a few hundred nanometers. The pristine cobalt oxide formed a nanorod-like morphology, as shown in ESI1. EDS spectra recorded for S1 and S2, shown in Fig. 1b, both indicated cobalt, oxygen and nickel as the main elements present, providing evidence that the cobalt oxide was successfully doped with nickel.

**Fig. 2:**
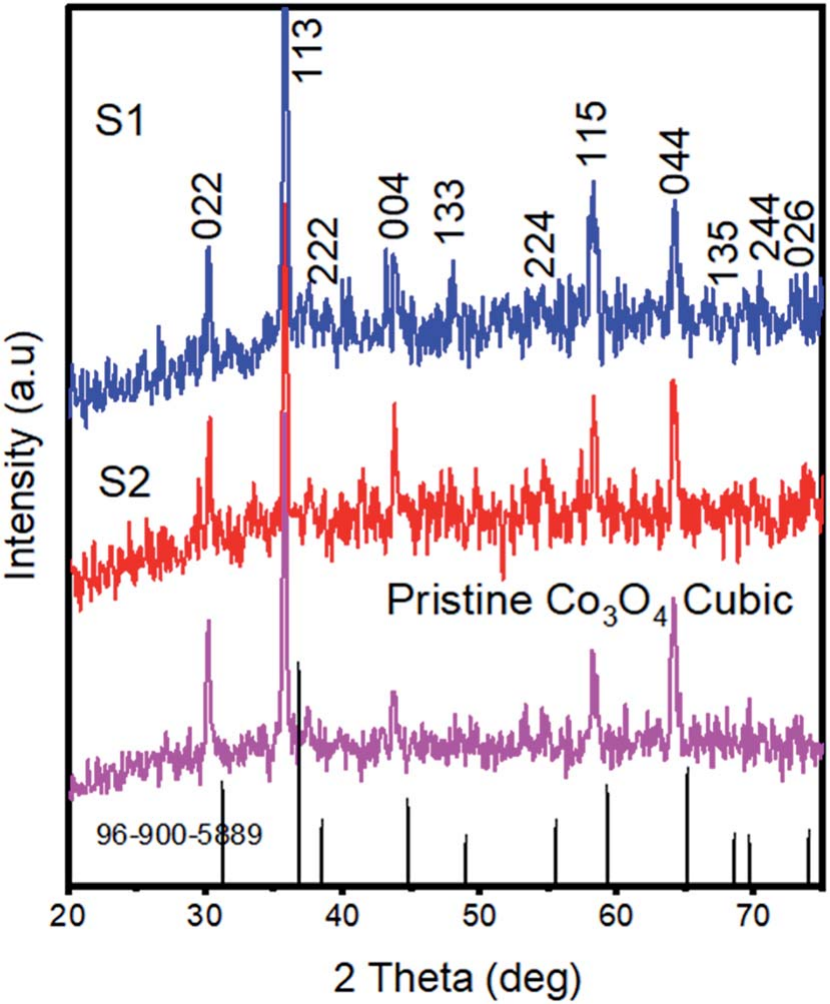
XRD patterns of pristine cobalt oxide, S1, and S2.

Powder XRD patterns were collected for pristine cobalt oxide, S1, and S2, as shown in Fig. 2. The diffraction patterns well matched the standard JCPDS card no = 96-900-5889, confirmed the cubic phase of cobalt oxide, and showed that the nickel doping did not alter the crystal structure of cobalt oxide. No other impurity related to a nickel phase was identified from the XRD analysis. Aggregated nanoparticles such as those we observed would be expected to facilitate charge transport and hence water oxidation due to the long-range continuity in the morphology and consequently more unavoidable contact with the electrolyte. The superior charge transport of S2 was further revealed by the EIS studies described below.

### 3.2. Electrochemical water oxidation using various nanostructured materials

**Fig. 3:**
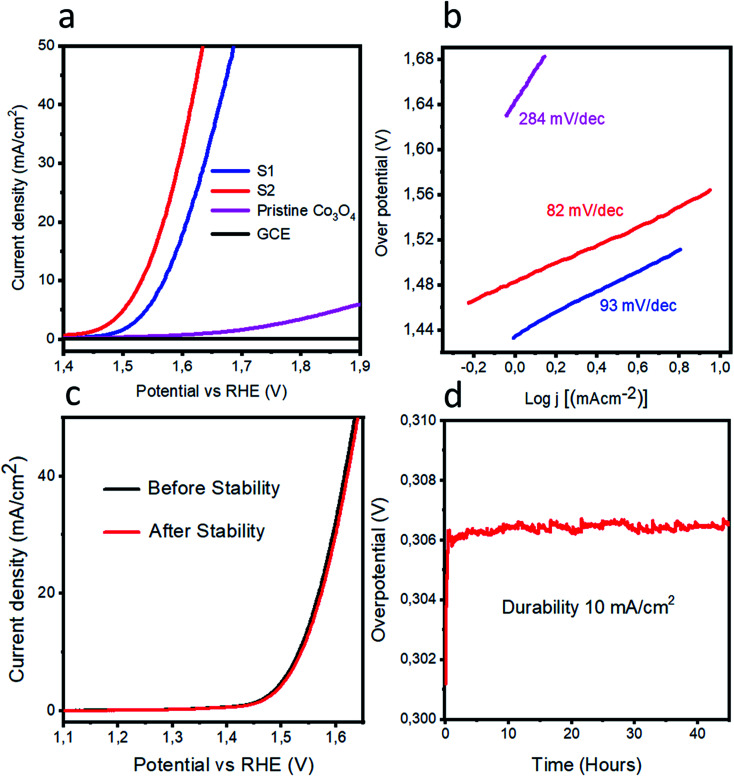
(a) LSV polarization curves of pristine cobalt oxide, S1, and S2, each at scan rate of 1 mV s^−1^ in 1 M KOH. (b) Tafel plots from the linear regions of the polarization curves. (c) LSV polarization curves for evaluating the stability of S2. (d) Chronopotentiometry experiment involving measuring the durability of the response of S2 over the course of 40 h at a current density of 10 mA cm^−2^.

The OER activities were measured for pristine cobalt oxide, S1, and S2 by performing LSV experiments each at a scan rate of 1 mV s^−1^ in a 1.0 M KOH solution. Prior to acquiring the LSV curve, several CV runs were carried out at a scan rate of 10 mV s^−1^ in order to stabilize the electrode. The LSV curves are shown in Fig. 3a. Pristine cobalt oxide showed poor OER activity with a high Tafel slope; this poor performance can be attributed to the poor conductivity and high charge-transfer resistance of pristine cobalt oxide. On other hand, an addition of nickel impurity increased the OER activity of cobalt oxide according to the data acquired for the S1 and S2 samples, also shown in Fig. 3a. We believe that the improved activity levels for these samples resulted from both their long-range morphologies and better crystalline structures as suggested by the SEM and XRD results. Their LSV polarization curves also showed reduced overpotentials for the OER, which revealed an enhanced conductivity of the doped cobalt oxide. Also, the doping might have altered the electronic states of the cobalt oxide, and thereby created oxygen vacancies within the cobalt oxide, leading to the observed enhanced OER. The S2 sample required an overpotential of 300 mV to reach a current density of 10 mA cm^−2^, far better than the results for the pristine cobalt oxide and recently reported nonprecious catalysts.^52–59^ Tafel plots were extracted from the linear regions of the LSV polarization curves, and the measured Tafel plot slope values for the pristine cobalt oxide, S1, and S2 samples were 284, 93 and 82 mV dec^−1^ as shown in Fig. 3b. The Tafel slope for S2 was found to be lower in value than those of the reported noble-metal-free catalysts.^52–59^ The pristine cobalt oxide exhibited a high Tafel slope value, due to its poor conductivity. The OER mechanism here was very difficult to illustrate due to its involving a four-electron-transfer process, but the most acceptable mechanism for OERs in alkaline medium for transition metal oxides has been reported to involve the equations^60–62^

1 M + OH^−^ → MOH + e^−^

2 MOH + OH^−^ → MO + H_2_O + e^−^

3 MO + OH^−^ → MOOH + e^−^

4 MOOH + OH^−^ → MO_2_ +*s*H_2_O + e^−^

and

5 MO_2_ → M + O_2_

where M indicates the active sites present within the catalyst and step (3) is the rate-determining step.

Stability and durability are two important parameters for the evaluation of catalyst performance, and they have been investigated for the S2 sample as shown in Fig. 3(c and d). Fig. 3c shows the stability curves before and after the durability test. A negligible loss of current density and a negligible shift in the OER onset potential were observed. Fig. 3d shows the durability experiment results obtained from a chronopotentiometry experiment carried out at a current density of 10 mA cm^−2^ for the S2 sample for 40 hours. The measured response was excellent and revealed the potential capability of S2 to retain the OER activity for a long period of time.

### 3.3. Electrochemical impedance spectroscopy (EIS)

**Fig. 4:**
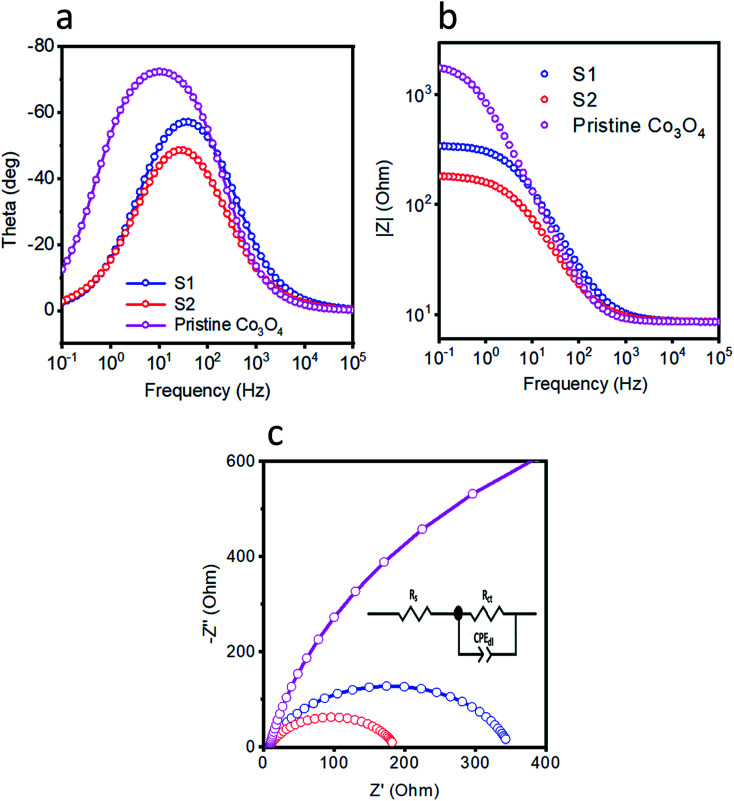
Electrochemical impedance spectra over the frequency range 100 kHz to 0.1 Hz, at an amplitude of 10 mV and onset potential of 1.53 V *vs.* RHE in 1 M KOH. (a and b) Bode plots. (c) Nyquist plots.

Electrochemical impedance spectroscopy (EIS) experiments were carried out to examine the charge-transfer characteristics of pristine cobalt oxide, S1, and S2. Fig. 4(a and b) show the corresponding Bode plots. The relaxation from the single peak in these plots can be attributed to charge-transfer kinetics. The Bode plot was used as another way to get in-depth information about the gain and phase features of the catalyst at various frequency regions. The phase angle showed the superiority of the OER activity of the S2 sample relative to those of the S1 and pristine cobalt oxide samples. Nyquist plots were recorded at 1.53 V *vs.* RHE as shown in Fig. 4c, and were used to calculate the resistance values. The impedance results were simulated using Z-view software and the fitted equivalent circuit model is shown as an inset in Fig. 4c. A solution resistance value of approximately 8 ohms was found for each sample. The charge-transfer resistance values of pristine cobalt oxide, S1, and S2 were estimated to be 1879, 339.9, and 177.5 ohms, respectively. The low charge-transfer resistance of S2 indicated its favorable OER kinetics, compared to those of S1 and pristine cobalt oxide. The fitted values for the equivalent circuit elements are given in Table 1. The addition of nickel into the cobalt oxide enhanced the charge transfer of S2 and in particular yielded swift transfers of electrons, and thus yielded the efficient OER activity. Double-layer capacitance values were also calculated from the impedance measurements for the pristine cobalt oxide, S1, and S2 samples, and were found to be 168.51, 122.20 and 232.68 mF, respectively, which also reflected the superior OER activity of S2. The low charge-transfer resistance and high double-layer capacitance of S2 ensured its favorable OER activity, and they were highly consistent with the acquired LSV polarization curves. The obtained results of low overpotential and Tafel slope of S2 were compared with the values for recently published OER catalysts in alkaline media^52–59^, as shown in Table 2. Compared to the onset potentials and durability levels of the reported catalysts, the newly developed catalyst showed a far better onset potential and comparable or better durability, respectively. The superior performance of the developed electrocatalyst can be attributed to the cobalt oxide containing the doped nickel, which might have acted as active sites and also further enhanced the conductivity of the material, thus resulting in the very good OER performance with low onset potential.

**Table 1:** The fitted values for equivalent circuit elements

	*R*_s_	*R*_ct_	*C*_dl_
Pristine Co_3_O_4_	8.65	1879	168.51
S1	8.61	339.9	122.20
S2	8.58	177.5	232.66

**Table 2:** Comparison of the presented OER catalyst with reported catalysts

Electrolyte	Catalyst	Tafel slope (mV dec^−1^)	Overpotential @ 10 mA cm^−2^	References
1 M NaOH	Co–P film	47	345	42
1 M KOH	CuCo_3_O_4_	60	—	43
1 M KOH	NiCo_3_O_4_	59	420	44
1 M KOH	CoCo LDH	59	393	45
1 M KOH	CoO_*x*_@CN	N/A	∼385	46
1 M KOH	MnCo_2_O_*x*_	84	>410	47
1 M KOH	Co_3_O_4_/N-rmGO	67	310	48
1 M KOH	NiCoO_*x*_	N/A	420	49
1 M KOH	N-G-CoO	71	340	50
1 M KOH	Ni-Co_3_O_4_	82	300	Present work

## 4. Conclusions

In summary, we successfully incorporated nickel dopant into cobalt oxide using a low-temperature aqueous chemical growth method. The analytical techniques showed the nanostructure and dimensions of the materials. The OER performances of the samples were evaluated in solutions of 1 M KOH, and sample S2 achieved a current density of 10 mA cm^−2^ at an overpotential of 300 mV, quite attractive for a nonprecious catalyst. S2 was found to be highly durable for 40 hours, having retained its OER activity over this time period. An EIS study showed a low charge-transfer resistance of 177.5 ohms for S2, lower than that for the pristine cobalt oxide sample, which indicated that the incorporated nickel enhanced the electron-transfer ability of the cobalt oxide. The attractive low overpotential, gradual Tafel slope, and low charge-transfer resistance values of the newly developed nickel-doped cobalt oxide catalyst suggested that this catalyst can be useful for a wide range of energy and environment-related applications.

## Conflicts of interest

Authors declare no conflict of interest in this research work.

## Supplementary Material

RA-010-D0RA00441C-s001
