# Supplementary material for: Facile doping of nickel into Co3O4 nanostructures to make them efficient for catalyzing the oxygen evolution reaction
Source: RSC Adv. 2020 Mar 31;10(22):12962–9. doi: 10.1039/d0ra00441c (PMC9051423; doi:10.1039/d0ra00441c)

Electronic Supplementary Material (ESI) for RSC Advances.
This journal is © The Royal Society of Chemistry 2020

Facile nickel doping into Co_3O_4 nanostructures turned them efficient for oxygen evolution reaction

Adeel Liaquat Bhatti[1], Umair Aftab[4], Aneela Tahira[2], Muhammad Ishaq Abro[4], Muhammad Kashif samoon[5], Muhammad Hassan Aghem[5], Muhamad Ali Bhatti[6], Zafar HussainIbupoto[3]*

[1]Institute of Physics, University of Sindh, Jamshoro, 76080, Sindh Pakistan

[2]Department of Science and Technology, Campus Norrköping, Linköping University, SE-60174 Norrköping, Sweden

[3]Institute of Chemistry, University of Sindh, 76080 Jamshoro, Pakistan.

[4]Mehran University of Engineering and Technology, 7680 Jamshoro, Sindh Pakistan

[5]Centre of Pure and Applied Geology University of Sindh, Jamshoro 76080, Sindh, Pakistan

[6]Centre of Environmental Sciences 76080, Jamshoro, Sindh Pakistan

*** Corresponding author:** Zafar Hussian Ibupoto, PhD

Email address: zaffar.ibhupoto@usindh.edu.pk

S1: SEM image of pristine cobalt oxide

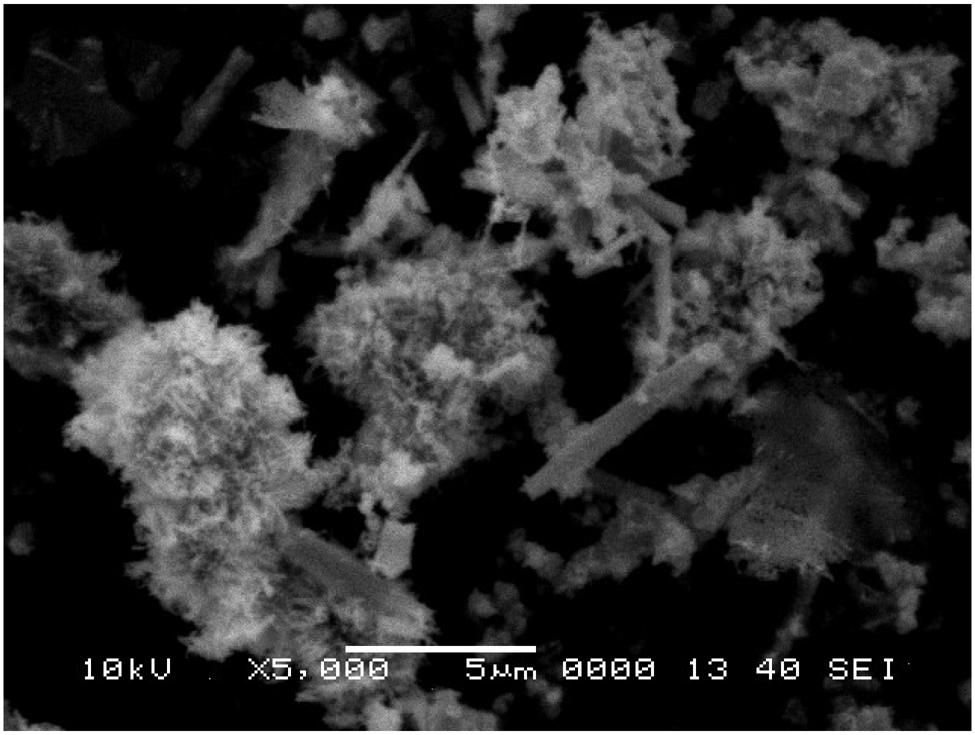

Supplement: RA-010-D0RA00441C-s001 [file RA-010-D0RA00441C-s001.pdf]
